# Hsa_circRNA_102051 regulates colorectal cancer proliferation and metastasis by mediating Notch pathway

**DOI:** 10.1186/s12935-023-03026-1

**Published:** 2023-10-05

**Authors:** Zhongsheng Chen, Haiyu Cheng, Jiandong Zhang, Dongbing Jiang, Gang Chen, Shunkang Yan, Wen Chen, Wei Zhan

**Affiliations:** 1 Guizhou Medical University, Guiyang, China; 2 Department of colorectal surgery, The Affiliated Hospital of Guizhou Medical University, No.28 Guiyi Street, Yunyan District, Guiyang City, 550004 Guizhou China

**Keywords:** Hsa_circRNA_102051, miR-203a, BPTF, Notch, Colorectal cancer

## Abstract

**Background:**

The purpose of this study was to investigate the role of hsa_circRNA_102051 in colorectal cancer (CRC) and its effect on the stemness of tumor cells.

**Methods:**

CircRNA microarray was under analysis to screen differentially expressed novel circRNAs in the pathology of CRC. Quantitative real-time PCR was used to detect the relative RNA expression in CRC cells and samples. The effects of hsa_circRNA_102051 on biological functions in CRC cells were accessed both in vitro and in vivo. FISH, RIP and luciferase reporter assay were conducted to confirm the regulatory correlations between hsa_circRNA_102051 and miR-203a, as well as miR-203a and BPTF. Xenograft models were applied to further verify the impacts and fluctuations of hsa_circRNA_102051/miR-203a/BPTF. Moreover, the mechanism how hsa_circRNA_102051 affected the Notch signals was also elucidated.

**Results:**

Hsa_circRNA_102051 was up-regulated in CRC tissues and cell lines, capable to promote the growth and invasion of CRC. In addition, hsa_circRNA_102051 could enhance stemness of CRC cells. BPTF was identified as downstream factors of hsa_circRNA_102051, and miR-203a was determined directly targeting both hsa_circRNA_102051 and BPTF as an intermediate regulator. Hsa_circRNA_102051 in CRC could block miR-203a expression, and subsequently activated BPTF. Hsa_circRNA_102051/miR-203a/BPTF axis modulated stemness of CRC cells by affecting Notch pathway.

**Conclusions:**

Our findings provided new clues that hsa_circRNA_102051 might be a potential predictive or prognostic factor in CRC, which induced the fluctuation of downstream miR-203a/BPTF, and subsequently influenced tumor growth, activities and stemness. Thereinto, the Notch signals were also involved. Hence, the hsa_circRNA_102051/miR-203a/BPTF axis could be further explored as a therapeutic target for anti-metastatic therapy in CRC patients.

**Supplementary Information:**

The online version contains supplementary material available at 10.1186/s12935-023-03026-1.

## Introduction

Colorectal cancer (CRC) is a malignancy that poses a significant challenge to the biotechnology field due to its high aggression and poor survival rates. Current data indicate that CRC has become leading cause of cancer deaths worldwide [1]. Whereas, the multifactorial pathobiology of CRC is still indistinct, for which cure therapies and early diagnosis remain limited [2, 3]. Therefore, further understanding in the occurrence and development of CRC diagnosis is urgent. Recent studies have shed light on the heterogeneous inner mechanisms of oncogenesis, revealing the role of specific genetic and epigenetic alterations in CRC development. In this study, we conducted an in-depth analysis of the driving factors involved in CRC cancerogenesis, with a particular focus on hsa_circRNA_102051. By exploring the genetic and epigenetic factors associated with CRC development, we hope to further our understanding of this complex disease and develop new biotechnological approaches for its diagnosis and treatment.

Previous reports have validated that circRNAs play a crucial role in various bioprocesses involved in malignancies by interacting with microRNAs and regulating transcription [4, 5]. Hsa_circRNA_102051 was one of the circRNAs influencing tumor progression. In esophageal squamous cancer, upregulation of hsa_circRNA_102051 was found to influencing downstream microRNAs and mRNAs [6]. Nevertheless, the expression and impacts of hsa_circRNA_102051 in CRC has never been reported. Here, this research discovered a novel pathway that hsa_circRNA_102051 could exert function by sponging miR-203a and subsequently modulate BPTF (Bromodomain PHD [plant homeodomain] Finger Transcription Factor) level in CRC cells and tissues. Our findings shed new light on the mechanisms involved in CRC development and provide a potential target for biotechnological interventions aimed at treating this deadly disease.

MiR-203a was proved to negatively affect multiple cancers, including CRC [7]. And bioprocesses of malignancies suppressed by miR-203a covered cell proliferation, migration, invasion, epithelial-mesenchymal transition (EMT) and so on [8–10]. According to circBank, hsa_circRNA_102051 is derived from TADA2A gene, which has been previously reported to produce circTADA2As capable of blocking miR-203a expression and influencing downstream factors [11, 12]. The correlation between hsa_circRNA_102051 and miR-203a was disclosed in this study, thereby highlighting the potential of circTADA2As as promising targets for biotechnological interventions in the cancer field.

In addition, BPTF was the downstream gene affected by hsa_circRNA_102051 and focused on in this paper. Being important in targeting the NURF (nucleosome remodeling factor) remodeling complex, BPTF was considered vital for the cell stemness [13]. Numerous studies have reported that BPTF could enhance self-renewal capacity of tumor-initiating cells (TIC) and cancer stem cells (CSC) [14, 15], thus promoting the progression and metastasis of malignancies, such as lung cancer and hepatocellular carcinoma (HCC) [16, 17]. Recent research has shown that BPTF is highly expressed in CRC and activated cell proliferation [18]. And another study we did showed that BPTF could be regulated by circRNAs in colon cancer, subsequently enhancing cell abilities [19]. Our current study has identified a novel regulatory mechanism of BPTF in CRC, shedding light on its impacts on the disease.

Briefly, this paper discovered the regulatory function of hsa_circRNA_102051 on miR-203a/BPTF expression, as well as downstream biological processes, especially the Notch signaling pathway, which composed a complete axis influencing CRC progression and metastasis.

## Method and material

### Clinical sample

The tumor tissues and matched adjacent tissues of 20 CRC patients admitted to the Affiliated Hospital of Guizhou Medical University from January 2018 to December 2019 were collected. All cases were confirmed by histopathological examination, and none of them received chemotherapy or radiotherapy before surgery. The study followed the Declaration of Helsinki, and all patients signed informed consent. Twenty samples of intestinal cancer tissues and adjacent tissues (normal colorectal mucosa located more than 5 cm away from the tumor margin, without invasion and avoiding tissues with inflammatory reactions) were collected in strict accordance with the collection standards during the operation. Some samples were frozen at -80℃, and some samples were fixed with 4% paraformaldehyde, dehydrated by automatic dehydrator, and preserved by paraffin embedding.

### Bioinformatics

GSE147597 chip was downloaded from the GEO database and then utilized to analyze circRNAs differentially-expressed in metastatic CRC, involving samples from 20 CRC tissues from patients with or without liver metastasis. Microarray analysis was performed using the software package “Limma” of R software (Ver. 3.6.3). The threshold was set to *P* value (corrected by Bonferroni - Holm) < 0.05, Fold Change > 2. Starbase and Circular RNA Interactome were respectively used to predict the miR-203a binding sites on hsa_circRNA_102051 and BPTF.

### Cell maintenance and transfection

Human CRC Cell lines including SW480, HT-29, SW1463 and HCT116, as well as FHCs (fetal human cells), were obtained from ATCC. SW480 and SW1463 cells were seeded in ATCC-formulated Leibovitz’s L-15 medium. HT-29 and HCT116 cells were cultured in ATCC-formulated McCoy’s 5a medium modified. FHCs were maintained in DMEM:F12 medium. All the cell lines were incubated under 37 °C with 5% CO_2_ in air atmosphere, and routinely subcultured every 3 days.

### Vector construction and cell transfection

Specific oligonucleotides and plasmids were designed to regulate the expression of hsa_circRNA_102051, miR- 203a, and BPTF. All the plasmids, siRNA, shRNA or mimics along with their corresponding negative controls, were designed and provided by GeneChem. The above oligonucleotides and plasmids were transfected into cell lines using a Lipofetamine 2000 transfection reagent according to the instructions. The expression efficiency was examined using quantitative PCR.

### Quantitative real-time PCR (qRT-PCR)

Total RNAs were extracted from CRC tissues and cells using Trizol reagent (Invitgen), and cDNA was then synthesized using a reverse transcription kit (Takara). In the ABI7900 system, cDNA amplification was performed using the Power SYBR Green (Takara) reaction mixture. The expression levels of hsa_circRNA_102051, miR-203a and BPTF were calculated by 2^−ΔΔCt^ with U6 or GAPDH as internal reference. All primer sequences are listed in Supplementary Table 1.

### Cell counting Kit-8 assay (CCK-8)

CCK-8 assay was performed to detect the viability of CRC cells (SW480 and HT-29 cell lines) with different transfections. Briefly, transfected cells were seeded in 96-well plates and supplemented with 10 µl CCK8 solution to each well at 0 h, 24 h, 48 h, and 72 h. After 2.5 h incubation, the cell viability was presented by the OD value at 450 nm under a microplate analyzer.

### RNase R

RNA was extracted from CRC cell lines using Trizol reagent (Invitrogen, USA). After that, 100 µg extracted RNA was incubated with RNase R at 37 °C for 20 min, and then purified using an RNEasy Minelute Cleanup Kit (Tokyo, Qiagen, Japan). Finally, the expression of RNA was detected through PCR.

### Immunohistochemistry (IHC) staining

All specimens were fixed in 4% formalin, embedded in paraffin and sectioned into 5 μm sections. Next, the sections were submerged in citrate buffer for antigen retrieval and incubated with 1% bovine serum albumin (BSA) to block nonspecific binding. Primary antibodies against Ki67 (1:500; ZSGB-BIO), BPTF (1: 500; ProMab) and appropriate secondary antibody (1:500, ZSGB-BIO) were utilized according to the manufacturer’s protocol. The sections were then incubated with DAB and hematoxylin and then scored independently by two observers. The score was based on both the proportion of positively stained tumor cells and the intensity of staining.

### Fluorescence in situ hybridization (FISH)

The digoxin-labeled probes specific to hsa_circRNA_102051 and biotin-labeled probes against miR-203a were prepared by Servicebio. SW480 and HT-29 cells were maintained on coverslips and fixed with 4% paraformaldehyde in PBS for 15 min. The probes were diluted in hybridization solution in PCR tubes and heated at 95 °C for 2 min in a PCR block to denature the probe. The probe was immediately chilled on ice to prevent reannealing. The hybridization solution was drained, and 100 µL of diluted probe per section was added to cover the entire sample. The samples were covered with a coverslip to prevent evaporation and were incubated in the humidified hybridization chamber at 65 °C overnight. The signals were detected by Cy3-conjugated anti-digoxin and FITC-conjugated anti-biotin antibodies (Jackson ImmunoResearch Inc.). Cell nuclei were counterstained with 4,6-diamidino-2-phenylindole (DAPI). The final images were obtained under a laser scanning confocal microscope (Nikon).

### EdU cell proliferation assay

24-well plates were coated with laminin (300 µL/well; Sigma) and incubated at least 4 h at 37 °C, then washed with PBS prior to cell seeding. Then, CRC cells in suspension were seeded manually at 2500 cells/well (5 cells/µL). Afterward, EdU staining assays were conducted to evaluate cell proliferation. Briefly, 10 µM 5-ethynyl-2′- deoxyuridine (EdU; Sigma) solution was added to each well and incubated for 3 h. After being washed with PBS, the cells were fixed in 4% paraformaldehyde and stained with EdU staining (Riobio) based on the manufacturer’s instructions. A fluorescence microscope (Leica DMI6000B) was utilized for eventual visualization and measurement.

### TUNEL fluorescence assay

According to the manufacture’s protocol, cells were firstly fixed with formaldehyde for 15 min on ice and then washed with PBS. After supplementation with ice-cold 70% ethanol and incubation for 30 min, cells were resuspended in wash buffer again. Staining solution was then added to the cells and maintained for 60 min at 37ºC. After another washing with rinse buffer, supernatant was discarded. The propidium iodide/RNAse A solution was applied for resuspending cells and 30 min incubation. And the eventual staining results were observed under a fluorescence microscope.

### Xenograft mice model

4-weeks-old female BALB/c nude mice were obtained from the Model Animal Research Center of Guizhou Medical University. For the establishment of CRC xenograft models, SW480 cells (3 × 10^6^) with different transfections were injected into the right flank of the nude mice. The tumor volumes were measured every 5 days using the formula: V = (length × width^2^)/2. After 25 days, mice were sacrificed, and tumors were collected, weighed and measured. All the animal experiments were performed under the guidelines of the Institution Animal Care and Use Committee.

### Western blot

Cells were lysed on ice with RIPA buffer (protein lysis buffer containing protease inhibitor and phosphatase inhibitor) for 30 min. And then the protein extracted from the supernatant was quantified through a BCA kit (Thermo Fisher). Then, the cell lysates were separated on SDS-PAGE and transferred to PVDF membranes. The membranes were blocked with 5% nonfat milk, and then incubated with primary antibodies at 4˚C overnight and with the secondary antibodies at room temperature for 2 h. Afterward, the target proteins were visualized with an enhanced chemiluminescence detection system.

### Dual-luciferase reporter assay

Dual luciferase reporter assays were performed to verify the binding sites between hsa_circRNA_102051 and miR-203a, as well as miR-203a and BPTF. Wild type or mutant sequences of above genetic factors were constructed using pmirGLO vectors (Promega). Cells were co-transfected with mimics or inhibitors, or their corresponding negative controls along with the luciferase reporter vector. After 48 h, luciferase activity of each system was measured using the dual luciferase reporter gene assay system (Promega) according to the manufacturer’s instructions.

### RNA-binding protein immunoprecipitation (RIP)

RIP experiments were carried out utilizing a Magna RIP RNA-Binding Protein Immunoprecipitation Kit (Millipore) following the manufacturer’s instructions. Cell lysates from SW480 and HT-29 were incubated with protein A magnetic beads conjugated to either antiAgo2 or IgG antibody. The coprecipitated RNA was purified and reversely transcribed into cDNA using PrimeScript RT Master Mix (TaKaRa Bio). The enrichment of hsa_circRNA_102051 and miR-203a pulled down by Ago2 or IgG from endogenous complex was then measured through quantitative PCR.

### Tumor sphere formation assays

A mixture culture medium was prepared including serum-free 1640 medium (Invitrogen), 2% B27 Supplement (Invitrogen), 20 ng/ml basal fibroblast growth factor (bFGF) (PeproTech), 20 ng/ml epidermal growth factor (EGF) (PeproTech), 0.4% BSA (Sigma-Aldrich), and 5 µg/ml insulin (Sigma-Aldrich). CRC cells were digested and resuspended at a density of 1000 cells/well into prepared medium. After being incubated at 37 °C and 5% CO_2_ with saturated humidity for 12–14 days, the tumor sphere was defined as > 2000 cells. The number of spheres divided by the original number of seeded cells was then counted and analyzed.

### Statistical analysis

All experiments were conducted at least three times, with the data presented as mean ± standard deviation. Statistical analysis was performed using GraphPad 7.0 (La Jolla). Differences between two groups were compared using t-test, and differences between multiple groups were compared using one-way analysis of variance (ANOVA), followed by Tukey-Kramer post-hoc analysis. *P* value less than 0.05 was considered statistically significant.

## Results

### Hsa_circRNA_102051 was identified as being overexpressed in metastatic CRC tissues through screening and analysis

**Fig. 1:**
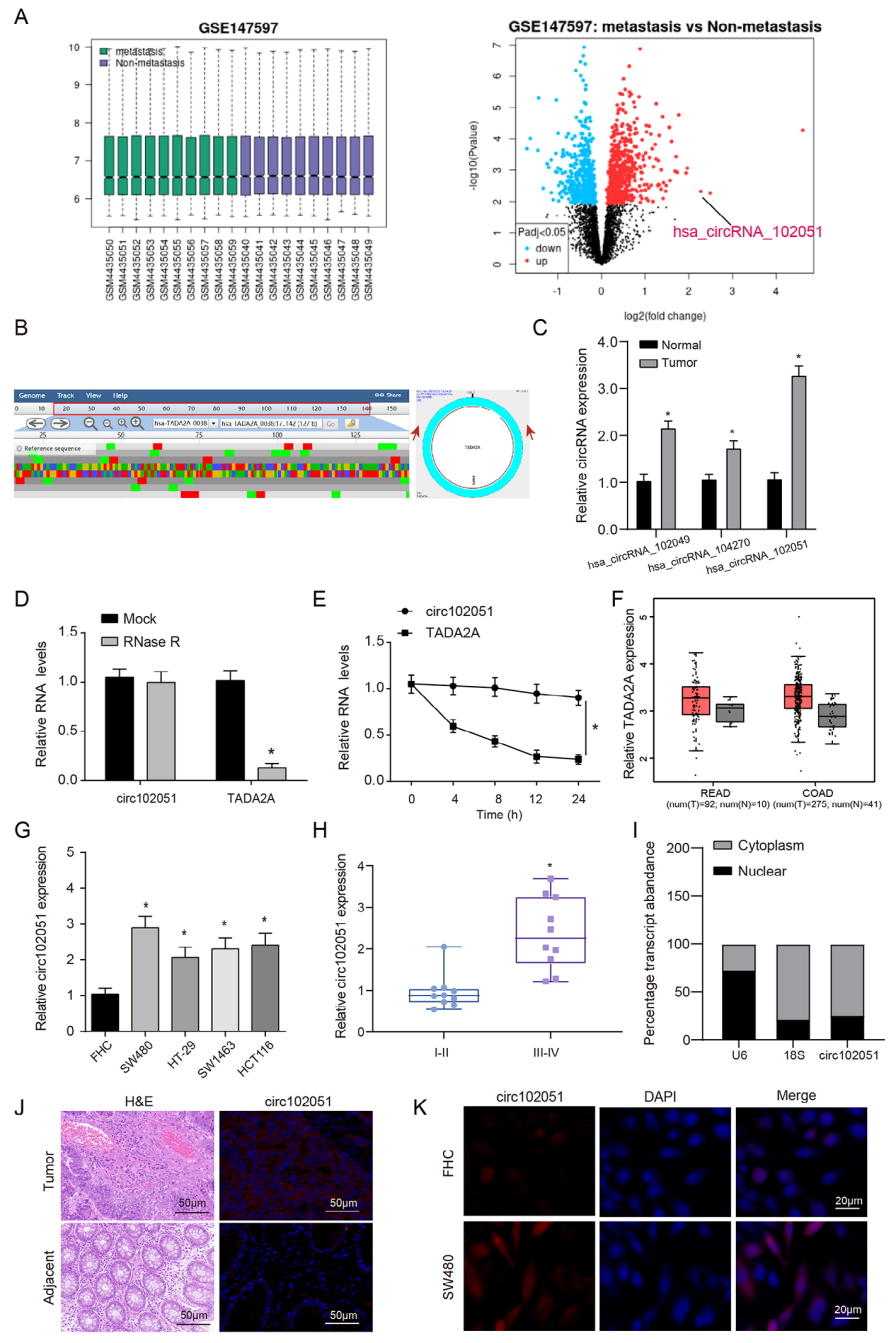
**Hsa_circRNA_102051 was overexpressed in metastatic CRC samples**. (**A**) GSE147597 was downloaded from the GEO database and processed difference analysis. 20 CRC samples from patients with or without liver metastasis were collected and detected. (**B**) Sequencing results showing the back-splice junction sequences of circ_102051. (**C**) Expression levels of candidate circRNAs in selected chips. Hsa_circRNA_102051 revealed the most significant overexpression in tumor samples compared to the normal ones. (**D**) RNase R identification of hsa_circRNA_102051. (**E**) RNA expression levels of hsa_circRNA_102051 and TADA2A along with time. TADA2A expression displayed a gradual drop while hsa_circRNA_102051 kept in high level. (**F**) TADA2A expression in colon and rectum adenocarcinoma tissues. TADA2A was overexpressed in colorectal tumors compared to normal tissues. (**G**) Expression of hsa_circRNA_102051 in CRC cell lines was higher than that in FHC cell line. (**H**) Expression of hsa_circRNA_102051 in tissues of Grade III-IV was higher than that in Grade 1-II. (**I**) QRT-PCR was used to detect the expression of has_circRNA_102051 in the nucleus and cytoplasm. (**J**) IHC staining of tumor and adjacent samples with corresponding hsa_circRNA_102051 expression. Hsa_circRNA_102051 was overexpressed in highly heterogeneous tumor tissues. (**K**) FISH testing of hsa_circRNA_102051 in FHC and SW480 cells. hsa_circRNA_102051 expression was mainly in cytoplasm and enhanced in SW480 cells. Compared to normal tissues or cells, ^*^P < 0.05

GSE147597 chip downloaded from the GEO database described the expression profiling of circRNAs in human CRC (Fig. 1A). Based on 20 CRC tissues from patients with or without liver metastasis, differentially-expressed circRNAs between two groups were visualized by volcano plot. Hsa_circ_102051, also known as hsa_circRNA_0006220, showed significant overexpression in metastatic tissue samples compared to non-metastatic samples. (Fig. 1A). The back-splice junction of circ_102051 was identified through sequencing, andthe gene symbol of hsa_circRNA_102051, TADA2A was located on chr17:35800605–35,800,763 (Fig. 1B). Expression of 3 candidate circRNAs in GSE147597, namely hsa_circRNA_102051, hsa_circ_102049 and hsa_circ_104270, was respectively detected and compared between tumor and normal tissues. Three candidates all displayed overexpression in tumor, among which hsa_circRNA_102051 showing the most prominent overexpression (Fig. 1C).

Therefore, hsa_circ_102051 was chosen for subsequent research and then identified through RNase R, indicating that TADA2A mRNA was digested while hsa_circRNA_102051 tolerated digestion (Fig. 1D). And TADA2A level was diving as time went on while hsa_circRNA_102051 kept a steady expression (Fig. 1E). Through the comparison between tumor and normal samples, TADA2A upregulation was determined in both colon and rectum adenocarcinoma (Fig. 1F). Four human colorectal adenocarcinoma cells (SW480, HT-29, SW1463, HCT116) were applied to further verification, and proved that hsa_circRNA_102051 overexpression existed in all above cell lines compared to FHC (Fig. 1G). Combined with clinical features, the detection on tissues of Grade I-II and Grade III-IV demonstrated that hsa_circRNA_102051 overexpression was enhanced in more advanced cases (Fig. 1H). In order to identify the sublocalization of has_circRNA_102051 cells, we isolated the nucleoplasm and detected that the circRNA was mainly distributed in the cytoplasm (Fig. 1I). IHC staining and FISH testing revealed that hsa_circRNA_102051 expression was enhanced in both tumor tissues and cells, with mainly distribution in the cytoplasm (Fig. 1J K).

### Hsa_circRNA_102051 promoted the proliferation, migration and invasion of CRC cells

**Fig. 2:**
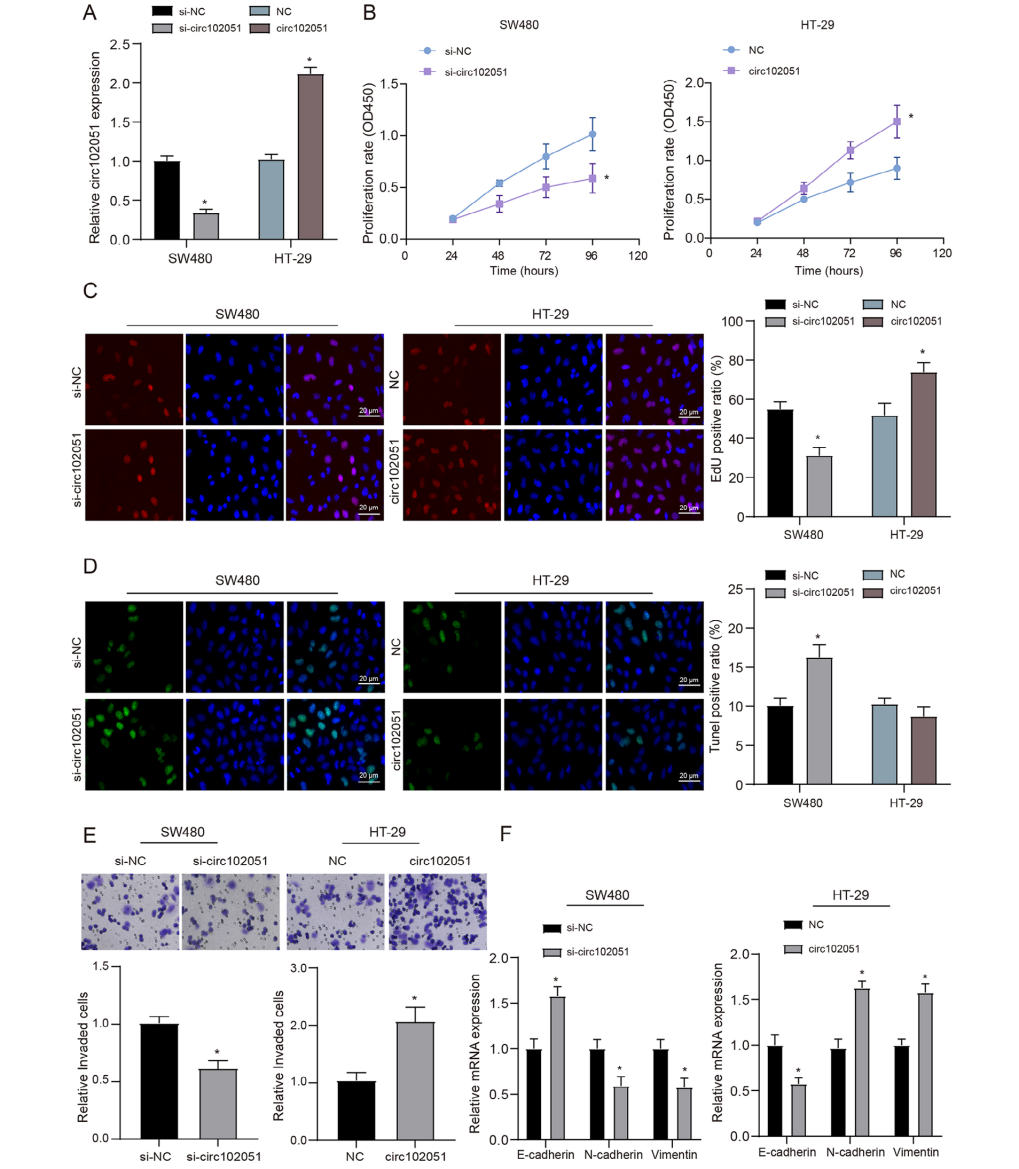
**Hsa_circRNA_102051 promoted the proliferation, migration and invasion of CRC cells**. (**A**) Transfection efficiency in SW480 and HT-29 cells. Expression of hsa_circRNA_102051 was inhibited with si-hsa_circRNA_102051 transfection in SW480 while enhanced with hsa_circRNA_102051 transfection in HT-29, compared to the si-NC or NC group. (**B**) CCK-8 assays in SW480 and HT-29 cells. (**C**) EdU cell proliferation testing in SW480 and HT-29 cells. Cell proliferation was inhibited in CRC cells when hsa_circRNA_102051 was downregulated while activated with additional hsa_circRNA_102051. (**D**) TUNEL fluorescence assay in SW480 and HT-29 cells. SW480 cells transfected with si-hsa_circRNA_102051 displayed a higher apoptosis rate, while apoptosis of HT-29 cells transfected with hsa_circRNA_102051 showed a decrease but the difference was not statistically significant. (**E**) Cell invasion in CRC cell lines was inhibited by si-hsa_circRNA_102051 while enhanced by hsa_circRNA_102051 transfection. (**F**) Expression of E-cadherin, N-cadherin and vimentin in SW480 and HT-29 cells. Invasion and migration of CRC cells were blocked by si-hsa_circRNA_102051 while enhanced by external hsa_circRNA_102051. Compared to si-NC or NC group, ^*^P < 0.05

CRC cell lines, SW480 and HT-29, were cultured in four groups. The first group of SW480 cells was transfected with si-NC, while the second group was transfected with si-hsa_circRNA_102051. The third group of HT-29 cells was transfected with vectors, and the fourth group was transfected with hsa_circRNA_102051. (Fig. 2A). The results from CCK-8 and EdU assays indicated that cell proliferation was suppressed in SW480 treated with si-hsa_circRNA_102051 while triggered in HT-29 treated with hsa_circRNA_102051 (Fig. 2B C), which proved the promoting effected of hsa_circRNA_102051 exerting on CRC cell multiplication. Cell apoptosis was detected and quantified by TUNEL fluorescence assay. SW480 cells transfected with si-hsa_circRNA_102051 displayed a higher apoptosis rate, while apoptosis of HT-29 cells transfected with hsa_circRNA_102051 showed a decrease but the difference was not statistically significant. This may be due to the low basal apoptosis rate (Fig. 2D).

After transfection of si-hsa_circRNA_102051, the number of gram invasive cells in SW480 cells decreased, while after transfection of hsa_circRNA_102051, the cell infiltration of HT-29 cell line increased (Fig. 2E). Correspondingly, cell migration in SW480 and HT-29 cells with different treatments was respectively assessed based on the expression of E-cadherin, N-cadherin and vimentin (Fig. 2F), indicating that CRC metastatic abilities were activated with upregulated hsa_circRNA_102051 while repressed by si-circRNA blockade. These findings demonstrate the potential of hsa_circRNA_102051 as a therapeutic target for controlling CRC metastasis.

### Hsa_circRNA_102051 was capable to promote tumor growth and metastasis in vivo

**Fig. 3:**
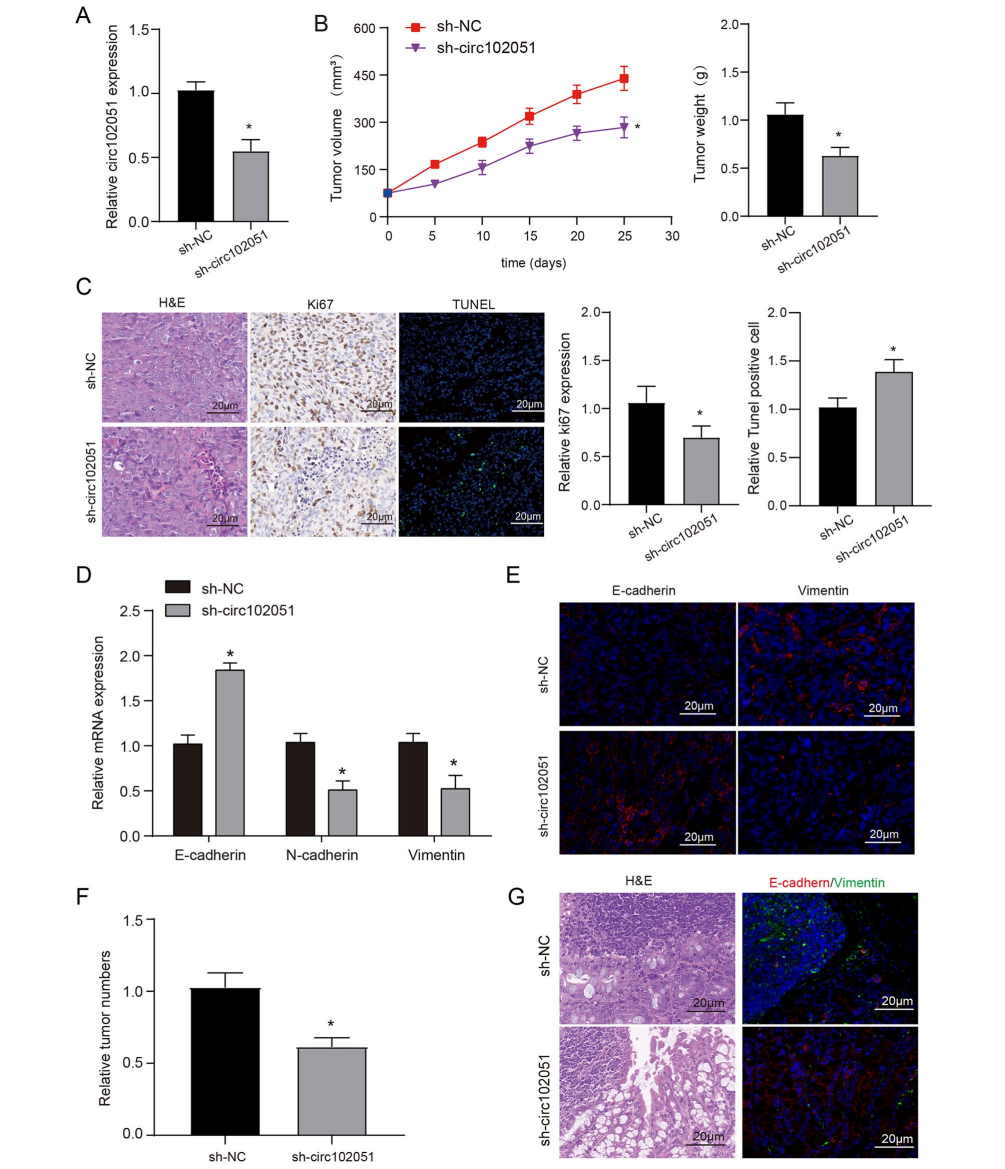
**Hsa_circRNA_102051 was capable to promote tumor growth and metastasis in vivo**. (**A**) Hsa_circRNA_102051 was successfully knocked down by sh-circ transfection in nude mice. (**B**) Tumor volume during 25 days and final weight in xenograft mice treated with sh-NC or sh-circ102051. Tumor growth was suppressed when hsa_circRNA_102051 was suppressed. (**C**) H&E staining, KI67 staining and TUNEL staining were used to verify the proliferation and apoptosis of tumor cells in different treatment groups (x200). (**D**) The mRNA expressions of E-cadherin, N-cadherin and vimetin in different tumor tissues were detected by QRT-PCR. (**E**) Immunofluorescence was used to detect the expression of E-cadherin and vimetin in the two groups of tumor tissues (x200). (**F**) Number of intestinal tumors and tumorigenesis in colorectal cancer induction model. (**G**) H&E and fluorescence staining of E-cadherin and vimentin in mouse tissues (x200). Compared to sh-NC group, ^*^P < 0.05

Xenograft mice were divided into two groups and separately treated with either sh-NC or sh-hsa_circRNA_102051 (Fig. 3A) The mice were then monitored for 25 days and tumor volume was measured every five days during the monitoring period. Tumor weight was detected after the mice were sacrificed. Results showed that in mice treated by sh-hsa_circRNA_102051, the growth of tumor volume was significantly slower and the eventual weight was lower compared to those treated by sh-NC (Fig. 3B). These findings suggest that hsa_circRNA_102051 plays a critical role in promoting tumor growth in vivo.

Proliferation of malignancies in mice were assessed by Ki-67 and TUNEL staining of tissue samples. Meanwhile, the expression of E-cadherin, N-cadherin and vimentin was also determined. With blockade of hsa_circRNA_102051, cell proliferation and invasion were both repressed, while apoptosis was promoted with blockade of hsa_circRNA_102051 (Fig. 3C-E). Further, we constructed a colorectal cancer model and found that inhibiting circ_102051 expression significantly reduced the number of intestinal tumors (Fig. 3F). Furthermore, we also used HE and immunofluorescence to detect tumor tissues and found that the proliferation ability of sh-circ102051 group was weakened and the metastasis process was inhibited (Fig. 3G).

### Hsa_circRNA_102051 enhanced stemness of CRC cells

**Fig. 4:**
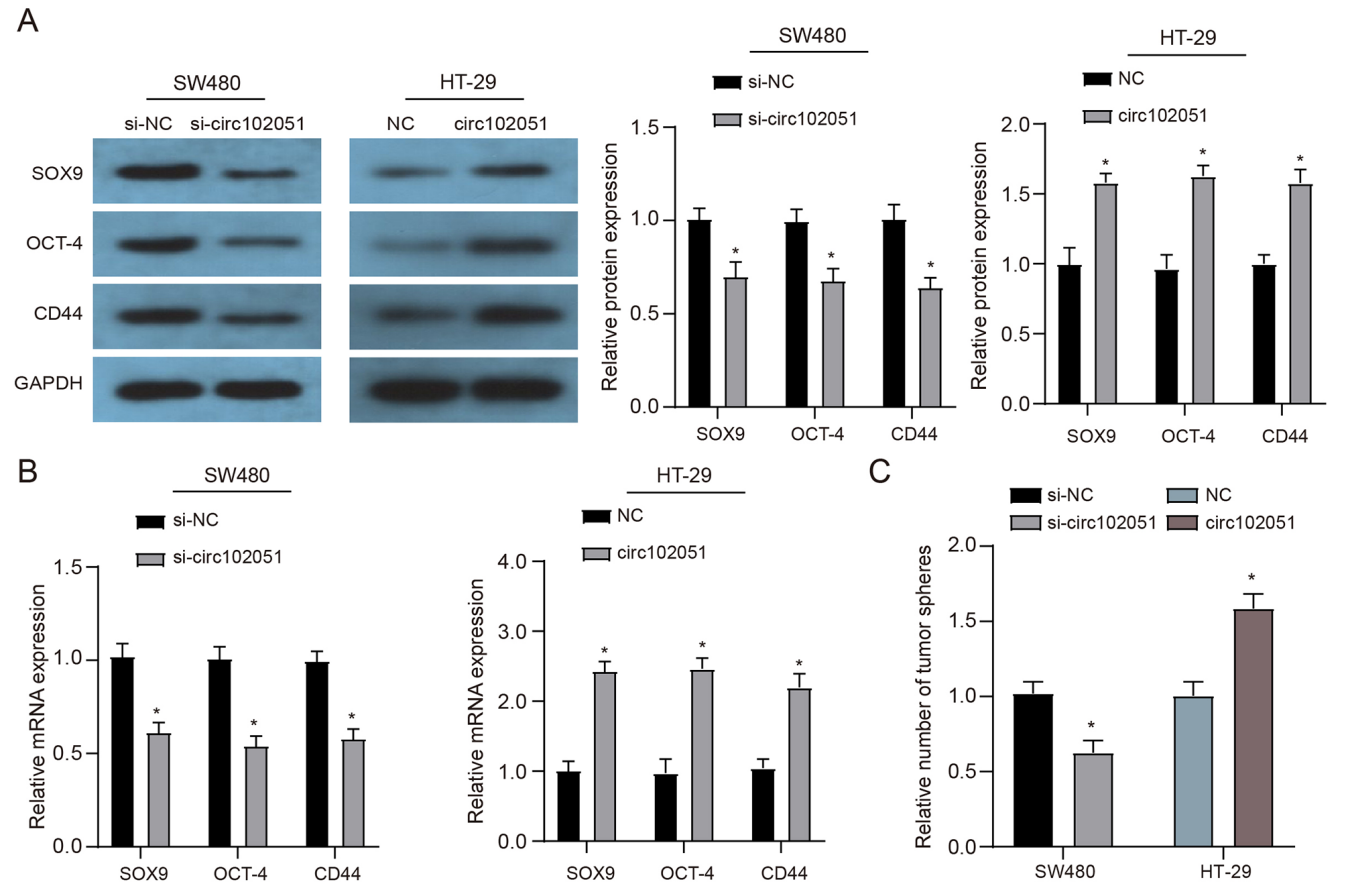
**Hsa_circRNA_102051 enhanced stemness of CRC cells**. (**A**) Western blot of SOX9, OCT-4 and CD44 in SW480 and HT-29 cells with different treatment. (**B**) QRT-PCR of SOX9, OCT-4 and CD44 in SW480 and HT-29 cells with different treatment.Expression levels of SOX9, OCT-4 and CD44 were all decreased by si-hsa_circRNA_102051 while increased by additional hsa_circRNA_102051. (**C**) In-vitro tumor sphere formation assays in four groups of SW480/HT-29 cells. Tumor formation was activated by hsa_circRNA_102051 while suppressed by si-circ treatment. Compared to NC or si-NC group, ^*^P < 0.05

SOX9, OCT-4 and CD44 are known to confer stemness properties in embryonic cells and several malignancies. Hence, this study measured their expression in SW480 and HT-29 cell lines to explore whether hsa_circRNA_102051 could influence cell stemness in CRC. SW480 cells were treated with si-NC or si-hsa_circRNA_102051, while HT-29 cells were treated with vector or hsa_circRNA_102051. According to outcomes of western blot and qRT-PCR, expressions of SOX9, OCT-4 and CD44 were all downregulated in si-circ-treated SW480 while upregulated in HT-29 cells transfected with hsa_circRNA_102051 (Fig. 4A and B). The in vitro sphere assay results demonstrated that si-circ-treated SW480 cells had the lowest ability to form tumor spheres, while HT-29 cells treated with additional hsa_circRNA_102051 had the highest ability to form tumor sphere (Fig. 4C). Therefore, Hsa_circRNA_102051 is considered to be an inducer to the stemness of CRC cells, and the enhanced stemness could be reversed by hsa_circRNA_102051 downregulation in vitro.

### BPTF was positively regulated by hsa_circRNA_102051 while negatively affected by miR-203a

**Fig. 5:**
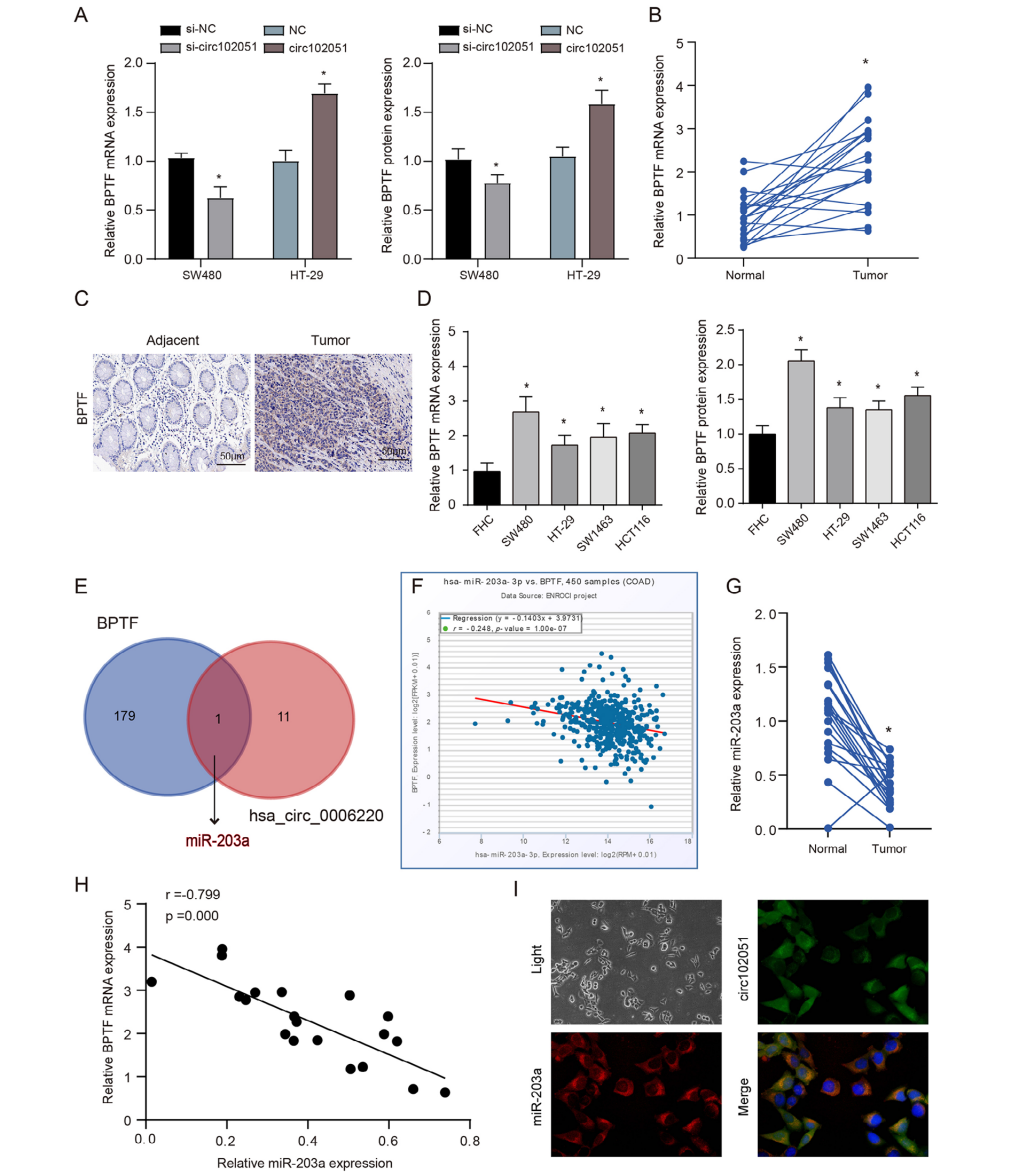
**BPTF was positively regulated by hsa_circRNA_102051 while negatively affected by miR-203a**. (**A**) mRNA and protein expression of BPTF in SW480 and HT-29 cells with different levels of hsa_circRNA_102051. The higher hsa_circRNA_102051 level is, the more expression BPTF displayed. (**B**) mRNA expression of BPTF in tumor and corresponding adjacent tissue. (**C**) IHC staining of BPTF in tumor and corresponding adjacent tissue. BPTF was overexpressed in CRC samples. (**D**) mRNA and protein expression of BPTF in FHC cells and four CRC cell lines (SW480, HT-29, SW1463, HCT116). BPTF was overexpressed in CRC cell lines. (**E**) miRNAs interacting with both hsa_circRNA_102051 and BPTF were predicted by Starbase and Circular RNA Interactome. miR-203a was the mediate factor between hsa_circRNA_102051 and BPTF. (**F**) Relation analysis based on COAD data. BPTF expression was positively correlative to miR-203a level, R = -0.248. (**G**) MiR-203a expression in tumor and corresponding adjacent tissue. MiR-203a was downregulated in CRC. (**H**) Relation analysis within tissue samples. BPTF expression was positively correlative to miR-203a level, R = -0.799. (**I**) FISH staining of miR-203a and hsa_circRNA_102051. MiR-203a and hsa_circRNA_102051 was co-located in cytoplasm of colorectal cells. Compared to adjacent samples or normal cells, ^*^P < 0.05

The previous studies have shown that BPTF has been prove to mediate the tumorigenesis, metastasis and relapse of TICs in colon via circRNA signals [19]. Therefore, this study aimed to investigate the interaction between BPTF and hsa_circRNA_102051 and their impact on cell stemness. Through detections on SW480 and HT-29 cells, BPTF expression showed a positive correlation to the level of hsa_circRNA_102051 in vitro (Fig. 5A). Furthermore, BPTF was found to be overexpressed in tumor samples compared to corresponding adjacent tissues (Fig. 5B C). This observation was further confirmed by comparing the expression of BPTF in CRC cell lines and normal cells at both transcriptional and translational levels (Fig. 5D).

Since BPTF was confirmed to be modulated by hsa_circRNA_102051 level, the mediate factor between hsa_circRNA_102051 and BPTF was predicted by Starbase and Circular RNA Interactome, and miR-203a was screened out (Fig. 5E). Relation analysis based on COAD data indicated that BPTF expression was positively correlative to miR-203a (Fig. 5F), which was further determined in clinical samples (Fig. 5H). The comparison between tumor and adjacent tissues identified that miR-203a was downregulated in CRC (Fig. 5G). Furthermore, FISH assays demonstrated that miR-203a was also enriched in cytoplasm, with the same location as hsa_circRNA_102051 (Fig. 5I).

### Hsa_circRNA_102051 downregulated miR-203a expression and subsequently activated BPTF

**Fig. 6:**
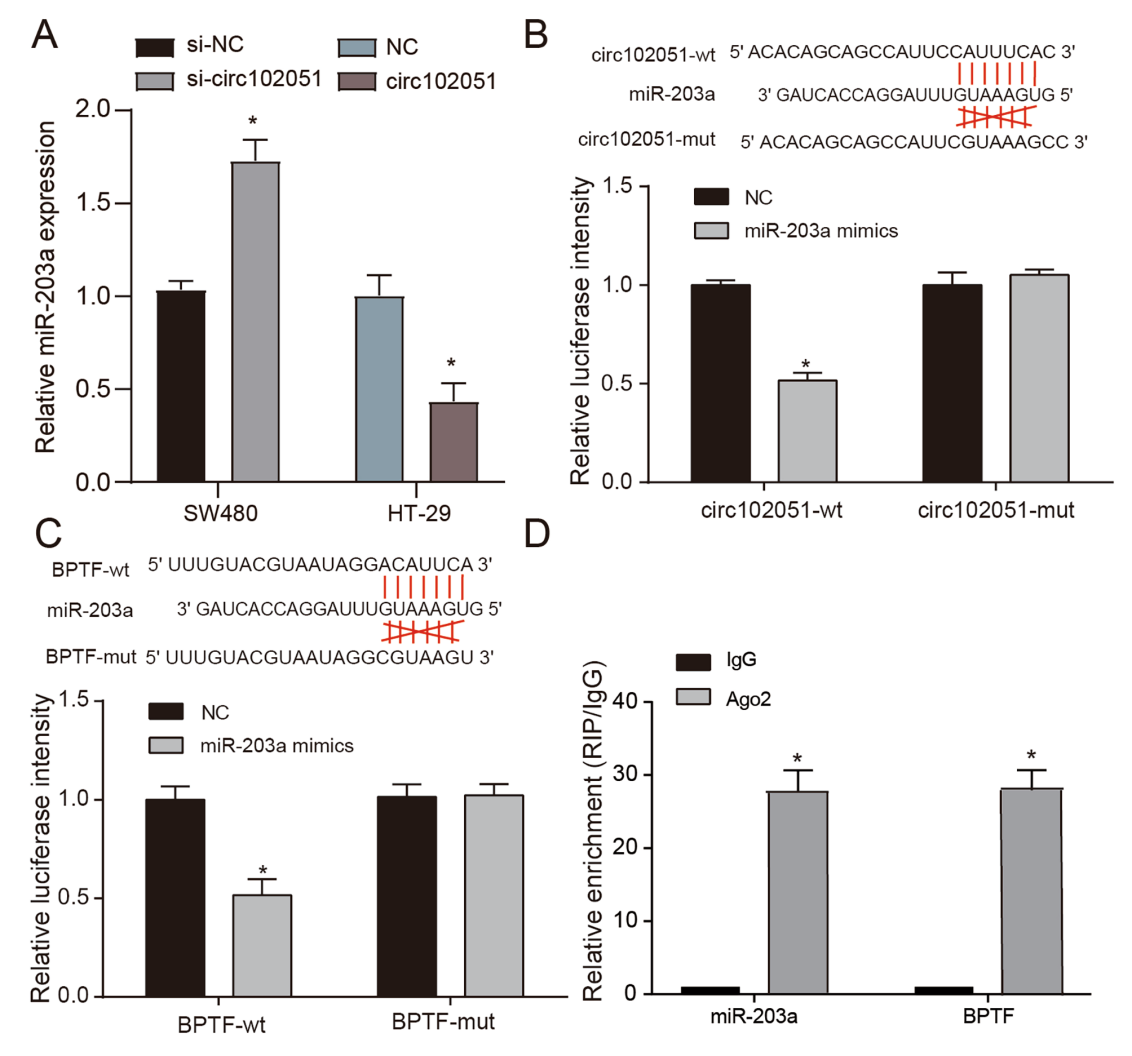
**Hsa_circRNA_102051 downregulated miR-203a expression and subsequently activated BPTF**. (**A**) MiR-203a expression in SW480 and HT-29 cells with different levels of hsa_circRNA_102051. High level hsa_circRNA_102051 triggered BPTF expression. (**B**) Dual luciferase assays revealed the binding targets between hsa_circRNA_102051 and miR-203a. (**C**) Dual luciferase assays displayed that miR-203a directly targeted BPTF. (**D**) Ago2 RIP of miR-203a and BPTF. MiR-203a and BPTF was significantly enriched. Compared to NC group, ^*^P < 0.05

According to the dataset of COAD cancer, patients with high miR-203a expression exhibited better overall survival than those with low level miR-203a (Supplementary Fig. 1A). Given that different level hsa_circRNA_102051 could regulated the miR-203a expression (Fig. 6A), this research did an in-depth exploration into the interactions between these three factors. The directly binding relation between hsa_circRNA_102051 and miR-203a was identified through dual luciferase assays, so was the binding target between miR-203a and BPTF (Fig. 6B C). Additionally, Ago2 RNA immunoprecipitation (RIP) was performed to isolate miR-203a and its target BPTF, which was significantly enriched from CRC cells (Fig. 6D). These results suggest that hsa_circRNA_102051 modulates the stemness of CRC cells by sponging miR-203a, which in turn regulates the expression of BPTF.

### Hsa_circRNA_102051 influenced CRC cell abilities via regulating miR-203a/BPTF

**Fig. 7:**
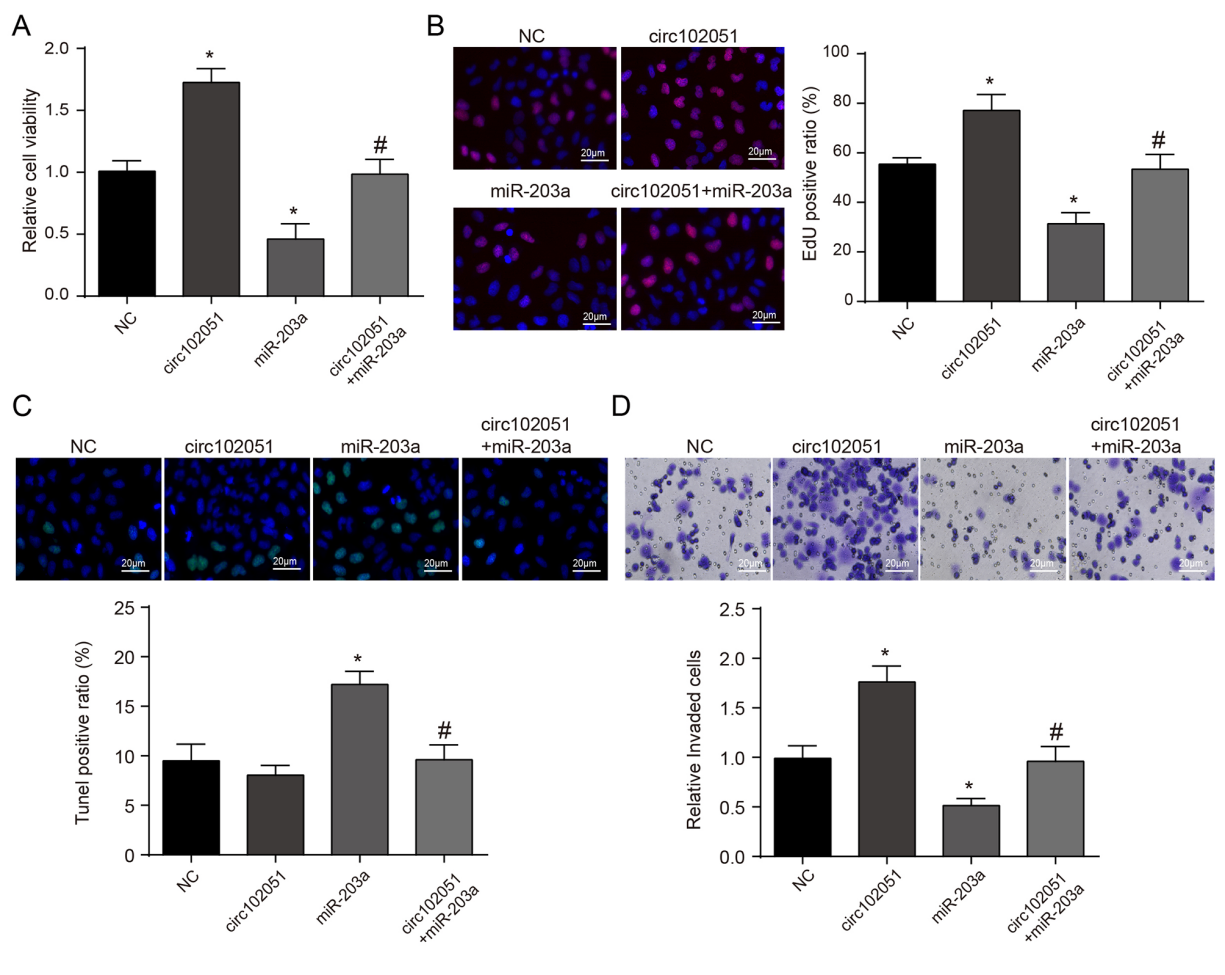
**Hsa_circRNA_102051 influenced CRC cell abilities via regulating miR-203a/BPTF**. (**A**) CCK-8 testing in CRC cells respectively transfected with vector, hsa_circRNA_102051, miR-203a, or hsa_circRNA_102051 plus miR-203a. Hsa_circRNA_102051 overexpression enhanced CRC cell viability while miR-203a inhibited it, which got reversed in cells treated with hsa_circRNA_102051 plus miR-203a. (**B**) EdU assays in CRC cells with different transfections. The proliferation of CRC cells was enhanced with additional hsa_circRNA_102051 while suppressed with miR-203a, and both were reversed in the hsa_circRNA_102051 plus miR-203a group. (**C**) TUNEL fluorescence assay in CRC cells with different treatments. The apoptosis of CRC cells was inhibited by hsa_circRNA_102051 while triggered by miR-203a, and both were reversed in those treated with hsa_circRNA_102051 plus miR-203a. (**D**) Cell invasion in CRC cell lines with different transfections. Cell invasion was promoted by hsa_circRNA_102051 while inhibited by miR-203a. The hsa_circRNA_102051 plus miR-203a group had similar invasion to the control. Compared to NC group, ^*^P < 0.05

To further investigate the regulatory mechanisms of hsa_circRNA_102051, miR-203a and BPTF, CRC cells were divided in to 4 groups, respectively transfected with vector, hsa_circRNA_102051, miR-203a, and hsa_circRNA_102051 plus miR-203a (Supplementary Fig. 1B-C). In vitro analysis demonstrated that cells with additional hsa_circRNA_102051 exhibited increased viability and proliferation, while those treated with miR-203a had weaken cell abilities. However, these effects were both reversed in the hsa_circRNA_102051 plus miR-203a group (Fig. 7A and B). The apoptosis of CRC cells was suppressed by hsa_circRNA_102051 while triggered by miR-203a. Once again, the combination of hsa_circRNA_102051 plus miR-203a reversed these effects (Fig. 7C). Cell invasion assays demonstrated that hsa_circRNA_102051 enhanced clone expansion, while miR-203a inhibited their invading ability. Whereas, CRC cells treated with hsa_circRNA_102051 plus miR-203a exhibited similar invasion to the control group (Fig. 7D).Overall, this study sheds light on the regulatory mechanisms of hsa_circRNA_102051 and miR-203a in CRC cell abilities. Hsa_circRNA_102051 promotes CRC development and progression by downregulating miR-203a expression. These findings provide new insights into potential targets for CRC diagnosis and therapy.

### Hsa_circRNA_102051/miR-203a/BPTF axis modulated stemness of CRC cells by affecting Notch pathway

**Fig. 8:**
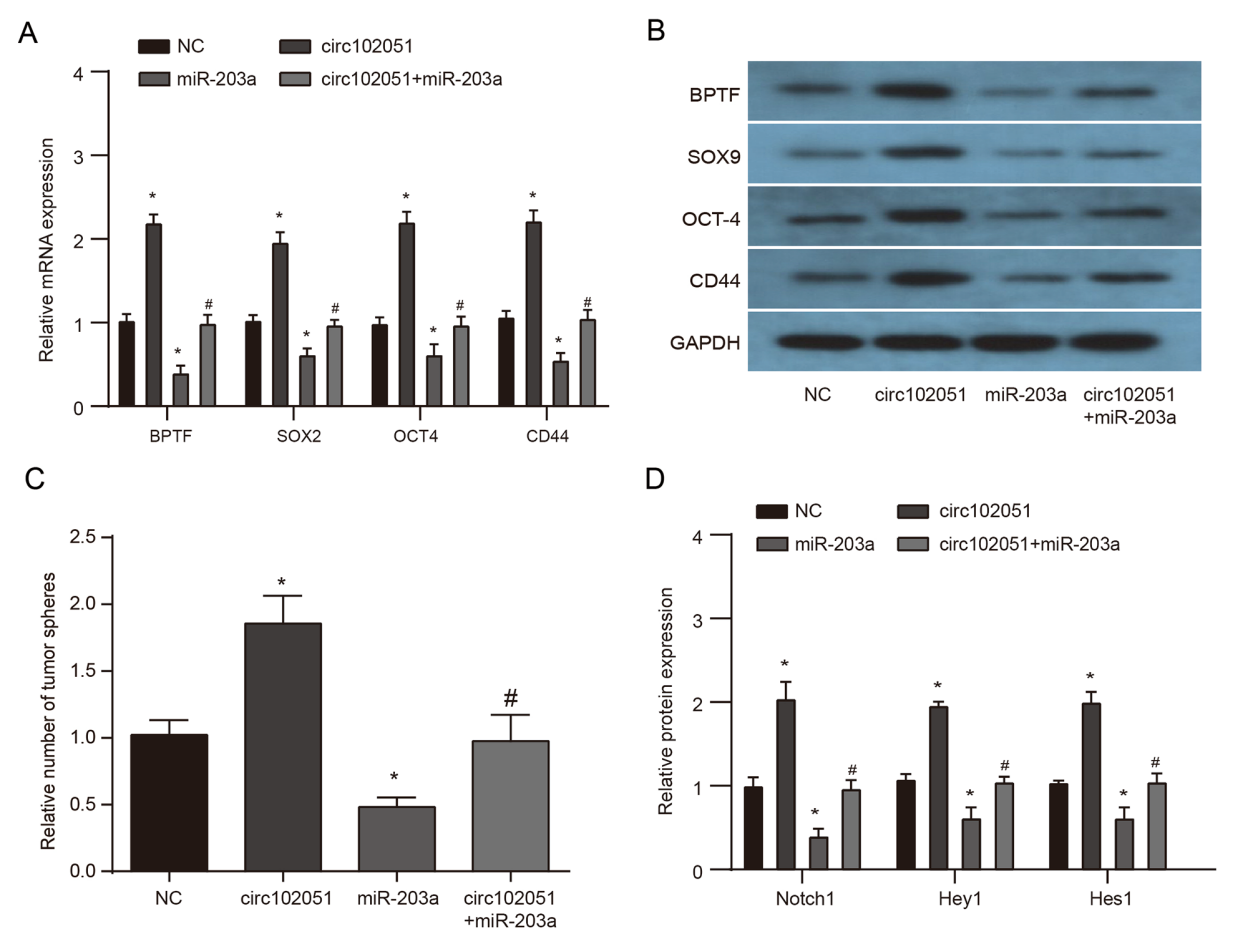
**Hsa_circRNA_102051/miR-203a/BPTF axis modulated stemness of CRC cells by affecting Notch pathway**. (**A**) qRT-PCR of SOX9, OCT-4 and CD44 in CRC cells transfected with vector, hsa_circRNA_102051, miR-203a, or hsa_circRNA_102051 plus miR-203a. (**B**) Western blot of SOX9, OCT-4 and CD44 in four groups of CRC cells. Expression levels of SOX9, OCT-4 and CD44 were all enhanced by hsa_circRNA_102051 while inhibited by miR-203a, which were reversed in the hsa_circRNA_102051 plus miR-203a group. (**C**) Tumor sphere formation assays in four groups of CRC cells. Tumor formation was activated by hsa_circRNA_102051 while suppressed by miR-203a treatment. The hsa_circRNA_102051 plus miR-203a group had similar number of tumor spheres to the negative control. (**D**) Western blot of Notch 1, Hey 1 and Hes 1 in CRC cells with different transfections. Notch signals were activated by hsa_circRNA_102051 while blocked by miR-203a, and were both reversed in the hsa_circRNA_102051 plus miR-203a group. Compared to NC group, ^*^P < 0.05

The stemness markers BPTF, SOX9, OCT-4 and CD44 were all detected in the four groups of CRC cells. According to the outcomes of qRT-PCR and western blot, expressions of BPTF, SOX9, OCT-4 and CD44 were all promoted by additional hsa_circRNA_102051 while repressed by miR-203a, which were reversed in the hsa_circRNA_102051 plus miR-203a group (Fig. 8A and B). In addition, *in-vitro* sphere formation indicated that hsa_circRNA_102051 promoted cell growth, while miR-203a inhibited it,and the hsa_circRNA_102051 plus miR-203a group generated a similar number of tumor spheres as the negative control (Fig. 8C). Moreover, Notch signaling proteins (Notch 1, Hey 1 and Hes 1) were found to be upregulated by hsa_circRNA_102051 but blocked by miR-203a, and these effects were both reversed in the hsa_circRNA_102051 plus miR-203a group (Fig. 8D). Therefore, the hsa_circRNA_102051/miR-203a/BPTF axis was capable to modulate the stemness of CRC cells via Notch signaling pathway, which subsequently influenced the process of cancerogenesis.

## Discussion

In this research, hsa_circRNA_102051 is screened out and identified with overexpression in metastatic CRC tissues. Upregulated hsa_circRNA_102051 is capable to suppress miR-203a and mediately trigger BPTF expression, which enhances the proliferation, migration, invasion and stemness of CRC cells, activating Notch signaling pathway and eventually promoting tumor growth and metastasis. Hsa_circRNA_102051/miR-203a/BPTF axis provided a novel angle of the modulation of CRC progression, and could be applied to clinic in future as predictive markers or therapeutic targets.

On the contrary to this study, previous researches on breast cancer reported that hsa_circRNA_102051 was downregulated in breast cancer patients, acting as a tumor suppressor and regulating miR-197-5p/CDH19 expression [20, 21]. As described before, hsa_circRNA_102051 in esophageal squamous cancer was also significantly upregulated, which is corresponding with the present research [6]. Therefore, the expression of hsa_circRNA_102051 could distinct within different categories of cancers. And hsa_circRNA_102051 could be capable to sponge various microRNAs besides miR-203a to exert other impacts, which deserves in-depth exploration.

As for miR-203a, abundant studies have its upregulation in CRC and several other cancers [7, 10, 22]. The epigenetic value of miR-203a as a predictive, prognostic, and targeting factor has been well documented. This study further confirmed the downregulation of miR-203a in CRC and determined its inhibiting effects on tumor progression. In addition, BPTF is another gene that has been shown to promote malignancy in several studies. Several studies display that BPTF exerts positive impacts on tumor through the MYC pathway [17, 23, 24], which is a promising signal for further monitoring.

In this study, stemness markers including BPTF, SOX9, OCT-4 and CD44 were all detected, indicating that overexpressed hsa_circRNA_102051 enhanced the stemness of CRC. Thereinto, SOX9 activation was proved to repress miR-203a transcription by binding to miR-203a promoter [25], while BPTF has been identified to promote HCC growth and enhance cancer stem cell traits [15]. Hence, investigating the underlying mechanisms of the interaction between stemness factors and miR-203a/BPTF axis could be a novel research direction in the future.

## Conclusion

Overall, this study identified hsa_circRNA_102051 as a key regulator of CRC progression and metastasis. The study demonstrated that hsa_circRNA_102051 promoted the expression of stemness markers including BPTF, SOX9, OCT-4, and CD44 through its ability to suppress miR-203a. This, in turn, activated the Notch signaling pathway, which promoted CRC growth and metastasis. The study highlights the potential of hsa_circRNA_102051 as a predictive biomarker or therapeutic target for CRC treatment. However, there are still unanswered questions, such as the inner mechanisms of the interaction between stemness factors and miR-203a/BPTF, which may be explored in future research.

### Electronic supplementary material

Below is the link to the electronic supplementary material.

Supplementary Material 1

Supplementary Material 2

Supplementary Material 3

## Data Availability

The datasets used and/or analyzed during the current study are available from the corresponding author on reasonable request.

## Abbreviations

CRC: Colorectal cancer
QRT-PCR: Real-time quantitative PCR
circRNA: Circular RNA
WB: Western blot
ceRNA: Competing endogenous RNA
IHC: Immunohistochemistry
H&E: Hematoxylin-eosin
ANOVA: Analysis of variance
