# Supplementary material for: Hsa_circRNA_102051 regulates colorectal cancer proliferation and metastasis by mediating Notch pathway
Source: Cancer Cell Int. 2023 Oct 5;23:230. doi: 10.1186/s12935-023-03026-1 (PMC10552285; doi:10.1186/s12935-023-03026-1)
Supplement: Supplementary file 1 — Supplementary Material 1 [file 12935_2023_3026_MOESM1_ESM.pdf]

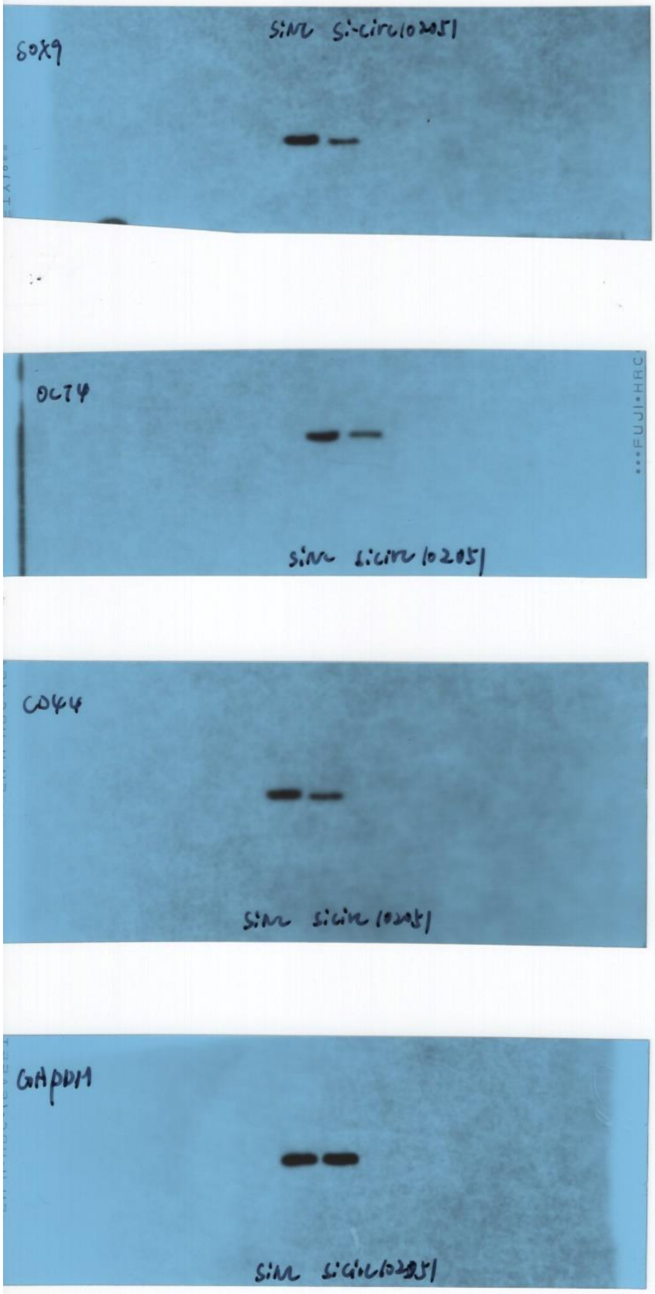

Figure4A（Full unedited blot）

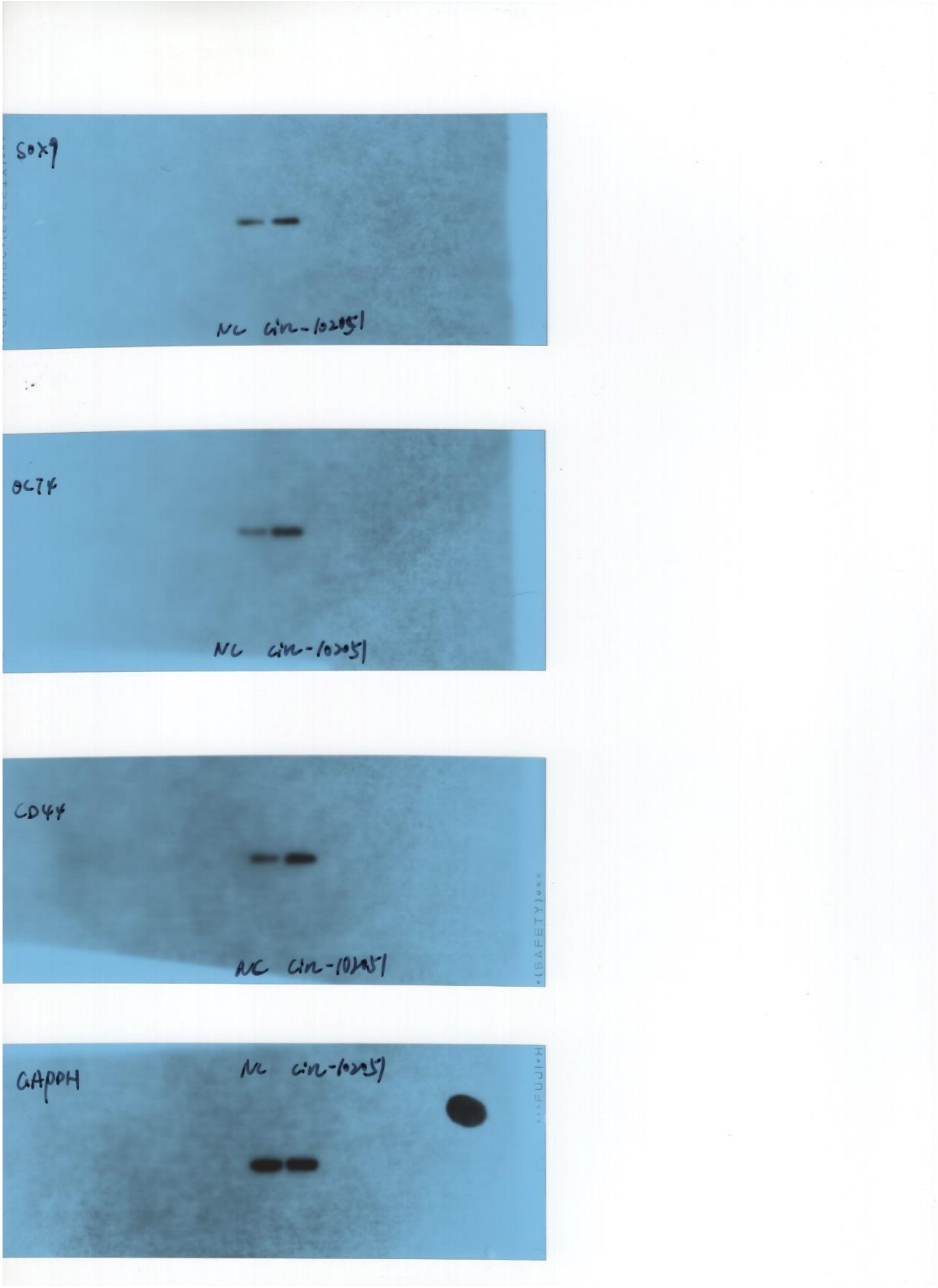

Figure4A（Full unedited blot）

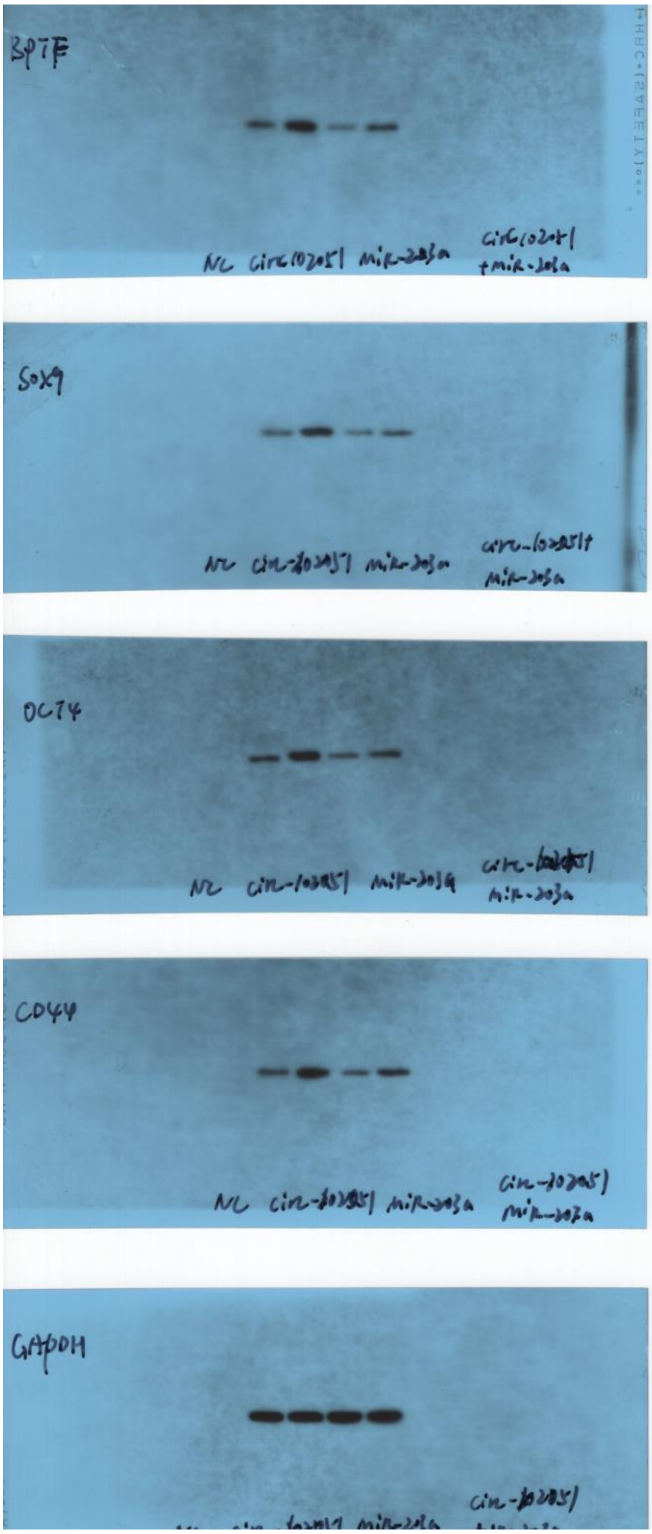

Figure8B（Full unedited blot）

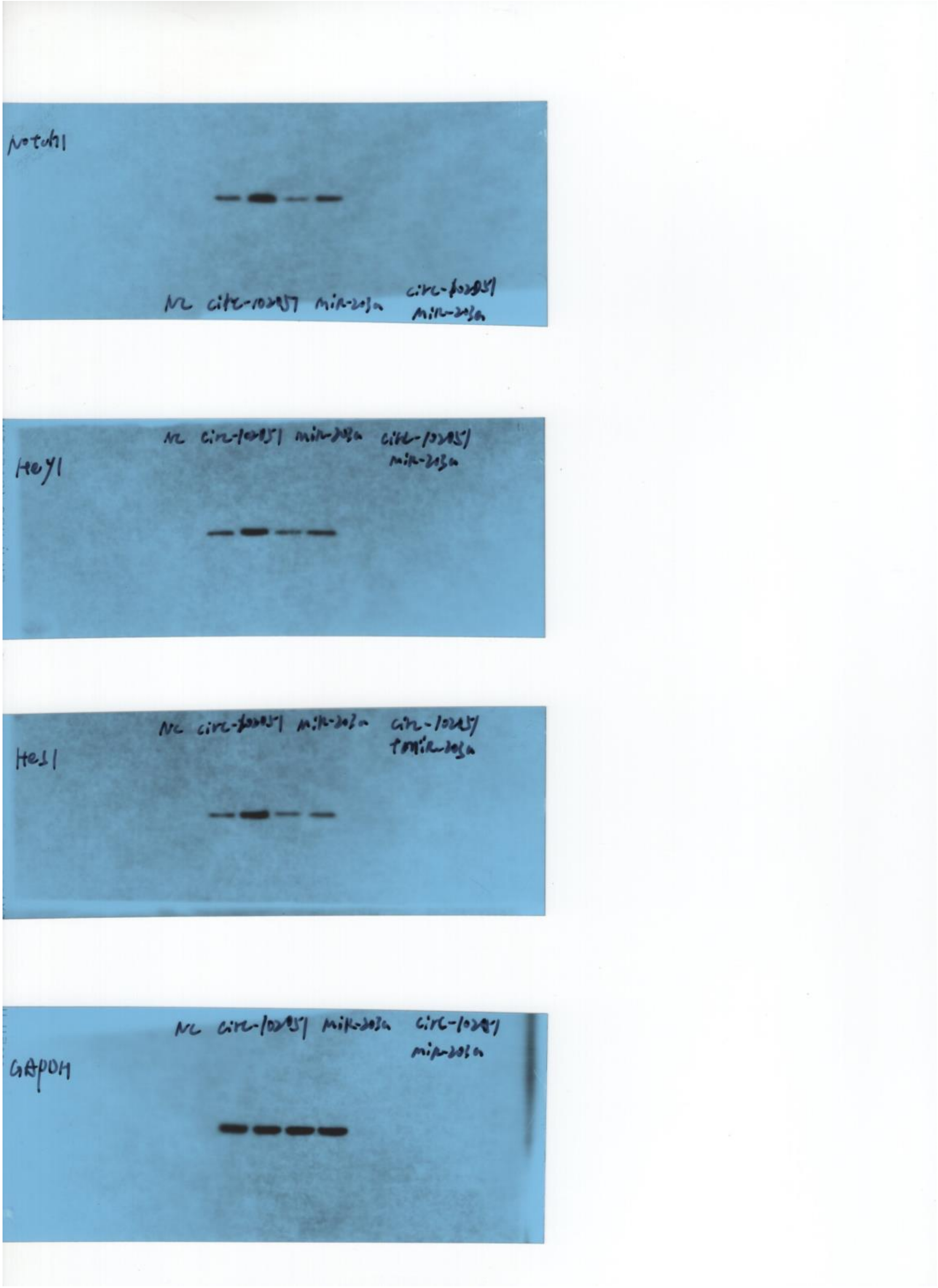

Figure8D（Full unedited blot）
