# Supplementary figures and images for: Hsa_circRNA_102051 regulates colorectal cancer proliferation and metastasis by mediating Notch pathway
Source: Cancer Cell Int. 2023 Oct 5;23:230. doi: 10.1186/s12935-023-03026-1 (PMC10552285; doi:10.1186/s12935-023-03026-1)

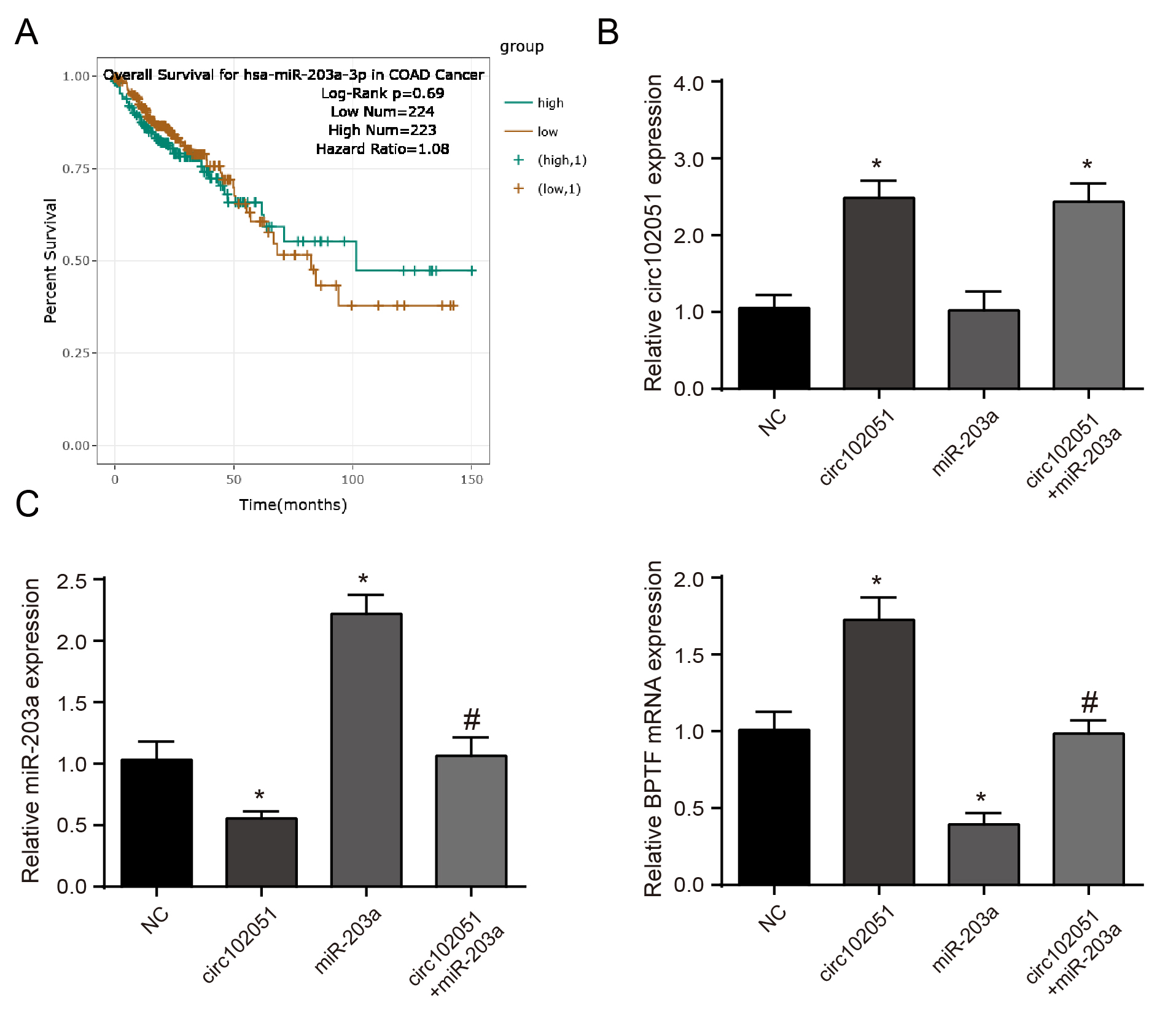

Supplement: Supplementary file 2 — Supplementary Material 2 [file 12935_2023_3026_MOESM2_ESM.jpg]
