# Supplementary material for: Hsa_circRNA_102051 regulates colorectal cancer proliferation and metastasis by mediating Notch pathway
Source: Cancer Cell Int. 2023 Oct 5;23:230. doi: 10.1186/s12935-023-03026-1 (PMC10552285; doi:10.1186/s12935-023-03026-1)
Supplement: Supplementary file 3 — Supplementary Material 3 [file 12935_2023_3026_MOESM3_ESM.docx]

**Table S1. Primer sequence**

| **Gene** | **Primer Sequence** |
| --- | --- |
| miR-203a forward | 5'- GTGAAATGTTTAGGACCACTAG-3 ' |
| U6 forward | 5’-ACAGATCTGTCGGTGTGGCAC-3’ |
| U6 reverse | 5’ -GGCCCCGGATTATCCGACATTC-3’ |
| SOX2 forward | 5’- ACCAGCTCGCAGACCTACA -3’ |
| SOX2 reverse | 5’- CGCGAGCCGAAAGTTTTGTA -3’ |
| CD44 forward | 5’- CCAATGCCTTTGATGGACC -3’ |
| CD44 reverse | 5’- GGACTTGACCACCGAAC -3’ |
| OCT-4 forward | 5’- GACAACAATGAGAACCTTCAGGAGA- -3’ |
| OCT-4 reverse | 5’- TTCTGGCGCCGGTTACAGAACCA- -3’ |
| BPTF forward | 5’- GAGGAGGAGGAGGAGGAGGAC -3’ |
| BPTF reverse | 5’- TCGTCGTCGTCGTCTTCCATC-3’ |
| E-cadherin forward | 5’-ATTTTTCCCTCGACACCCGAT-3’ |
| E-cadherin reverse | 5’-TCCCAGGCGTAGACCAAGA-3’ |
| N-cadherin forward | 5’-GAGAGGAAGACCA-GGACTATGA-3’ |
| N-cadherin reverse | 5’-CAGTCATCACCACCACCATAC-3’ |
| Vimentin forward | 5’-AGTCCACTGAGTACCGGAGAC-3’ |
| Vimentin reverse | 5’-CATTTCACGCATCTGGCGTTC-3’ |
| Circ_102051 forward | 5’- TGTTTGCATCTACCCTGCTG -3’ |
| Circ_102051 reverse | 5’- CACTCCTCCTTGGTCTTGGT-3’ |
| GAPDH forward | 5’- ACCCACTCCTCCACCTTTGAC-3’ |
| GAPDH reverse | 5’- TCCACCACCCTGTTGCTGTAG -3’ |
